# Metal Nanoparticles as Sustainable Tools for C–N Bond Formation via C–H Activation

**DOI:** 10.3390/molecules26134106

**Published:** 2021-07-05

**Authors:** Federica Valentini, Oriana Piermatti, Luigi Vaccaro

**Affiliations:** Laboratory of Green S.O.C., Dipartimento di Chimica, Biologia e Biotecnologie, Università degli Studi di Perugia, Via Elce di Sotto 8, 06123 Perugia, Italy; federicavalentinimail@gmail.com (F.V.); oriana.piermatti@unipg.it (O.P.)

**Keywords:** heterogeneous catalysis, metal nanoparticles, C–N bond formation, C–H activation

## Abstract

The design of highly active metal nanoparticles to be employed as efficient heterogeneous catalysts is a key tool for the construction of complex organic molecules and the minimization of their environmental costs. The formation of novel C–N bonds via C–H activation is an effective atom-economical strategy to access high value materials in pharmaceuticals, polymers, and natural product production. In this contribution, the literature of the last ten years on the use of metal nanoparticles in the processes involving direct C–N bond formation will be discussed. Where possible, a discussion on the role and influence of the support used for the immobilization and/or the metal chosen is reported. Particular attention was given to the description of the experiments performed to elucidate the active mechanism.

## 1. Introduction

Nowadays the development of efficient synthetic strategies to access value-added products in a cost-effective and atom-economical manner is an urgent challenge for modern chemistry. In the context of sustainable chemical production, the use of catalytic methods is therefore of key importance to access reactivity and selectivity that is otherwise inaccessible [1,2].

An effective step-economical catalytic construction of complex organic molecules is based on the use of direct C–H functionalization technologies [3,4,5,6]. Avoiding the needs of pre-functionalization substrates, the C–H functionalization methodology offers an effective approach to minimize the waste associated to the preparation of highly reactive building blocks [3,4,5,6,7,8,9,10,11,12,13,14]. The formation of C–metal bonds consecutive to the catalytic C–H bond cleavage increases the reactivity of otherwise inert C–H bonds, steering direct catalytic functionalization [15,16,17].

In this scenario, several efforts have been dedicated to the development of various transition metal-based catalysts. Although homogeneous metal complexes are highly efficient in C–H activation reactions [18,19,20,21,22,23,24,25,26], their difficult reusability and the inevitable waste produced from their separation from reaction products constitutes an important limitation for their actual use. To overcome these issues and improve the sustainability of C–H functionalization reactions, the design and use of heterogeneous catalysis is a valid yet challenging option [27,28,29,30,31,32,33,34,35,36,37,38].

Nanocatalysis have emerged as a well-defined subclass of heterogeneous catalysis that comprehend both colloidal and supported nanoparticles [39,40,41,42,43]. Tailor-made supports and ligands used for the stabilization of metal nanoparticles (MNPs) play a key role in the design of nanocatalysts, influencing both shape and size. The reduced metal dimension at nanoscale makes MNPs potentially highly effective and desirable catalysts. The peculiar properties of nanometric-sized materials make MNPs interesting for their applications in the development of sustainable protocols for C–H functionalization reactions. In fact, the use of rare precious metals, generally employed in homogeneous catalysis, can be minimized with increased activity at the nanoscale. Indeed, less-reactive non-noble metals also lead to efficient nanocatalytic systems or supports by enhancing their catalytic efficiency. Nanomaterials may also be endowed with specific additional properties that enhance their applicative interest. For example, when MNPs are employed as photocatalysts [44,45], light-activation can promote the single electron transfer mechanism (SET), or alternatively, NPs with magnetic properties can be separated from the reaction mixture, simplifying product isolation [46,47,48,49,50,51,52,53].

Catalyst design strictly influences the operative mechanism of heterogeneous nanocatalysts, and therefore may affect their recoverability and reusability in addition to the metal contamination in the products [35,36,37,38]. Indeed, when MNPs are used in catalysis, the catalytic cycle can take place directly on the nanoparticle surfaces (heterogeneous mechanism) or it can be promoted by some soluble species released in solution (homogeneous mechanism). Generally, in the latter case, effective nanocatalysts are of major interest if the metallic active species returns to the surface at the end of the catalytic cycle, defining a “release and catch” mechanism [54,55].

Among the possible transformation of the C–H bond, the direct formation of C–N bond is of great interest in pharmaceuticals, polymers, and natural products [56,57,58,59,60].

In this contribution, C–N bond formation via C–H activation processes catalyzed by metal nanoparticles will be discussed, the protocols are divided into C–H amidation, C–H amination, and C–N bond formation in heterocycle synthesis. In the processes described the use of cheap 3d metal NPs or their oxides are predominant. Only the synthesis of heterocycles is mostly confined to the employment of Pd-based nanomaterials.

In this literature survey, the selected examples also report data from investigations of the operative mechanisms. These data generally highlight the importance and the role of the support chosen and/or the conditions selected for the synthesis of MNPs to obtain high catalytic efficiencies.

## 2. C–H Amidation

**Scheme 1:**
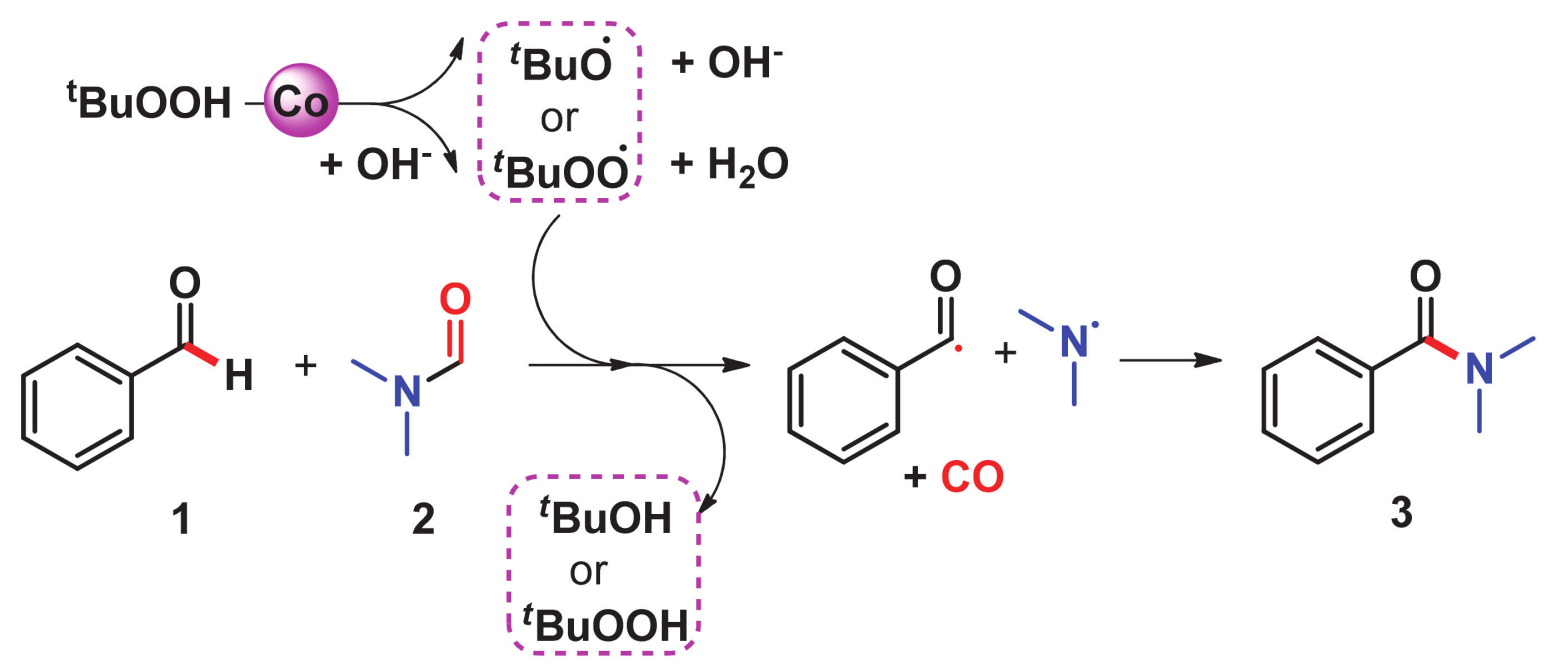
C–H amidation of aromatic aldehydes catalyzed by Co@C-N_600_.

In 2015, Li and coworkers proposed a synthetic strategy for the synthesis of amide **3** using Co NPs embedded in an *N*-doped carbon matrix (Co@C-N) [61]. The authors tested the influence of pyrolysis temperature on the catalytic activity of Co@C-N in the C–H amidation of aromatic aldehydes **1** with the amides **2** (Scheme 1). With this approach, several catalysts were obtained from pyrolysis of Co containing MOFs and compared with various homogeneous and heterogeneous catalysts. The authors found that the best catalytic system was obtained at 600 °C and, more importantly, the synergy between Co NPs and the N-doped carbonaceous matrix seems to be crucial. Among the oxidants tested, the best conditions were found when using 5 eq of *tert*-butyl hydroperoxide (TBHP) while the increment of oxidants leads to the formation of *p*-toluic acid as side-product. Several analyses have been executed in order to determine the heterogeneous nature of the catalytic process. The catalyst was easily separated from the reaction mixture using and external magnetic field and showed good recyclability for up to five consecutive runs. After a hot filtration test, less than 0.1% of metal was detected in solution. In addition, TEM analysis after the recovery of the material showed no aggregation of NPs. Furthermore, mechanistic investigation confirmed that the carbonyl in the product came from aldehydes, and the mechanism involved the formation of radicals. Indeed, in the experiment conducted in the presence of TEMPO, no further conversion was observed. After the reaction with ^13^C-DMF was carried out, the authors confirmed that the amidation process occurs via C–H activation.

The majority of the protocols developed for the synthesis of *N*-substituted amides are based on the use of copper or copper oxide nanoparticles. 

**Scheme 2:**
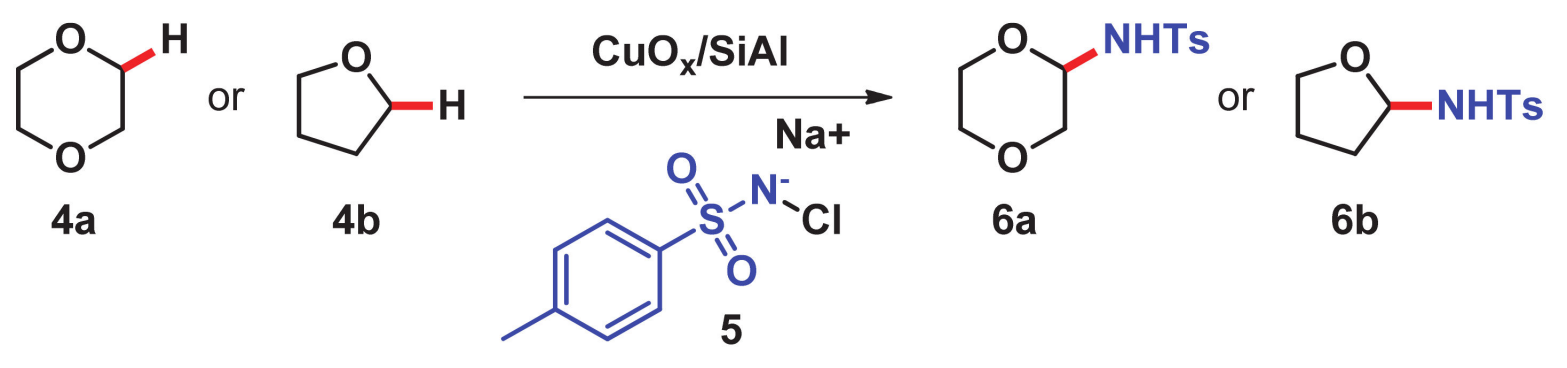
C–H amidation of **4** catalyzed by CuO/SiAl nanocatalyst.

In 2013, Biffis and coworkers examined the influence of different supports on the catalytic activity of CuO_x_ NPs in the C–H amidation of 1,4-dioxane **4a** or tetrahydrofuran **4b** with chloramine T **5** (Scheme 2) [62]. The authors exploited the importance of the surface area and pore volume of the supports in the stabilization of NPs and in their accessibility. In addition, the synthetic strategy for the synthesis of final catalysts allowed different Cu species on the supports. The authors found that the presence of Cu(I) was crucial for the activity of the nanocatalyst. Indeed, the best efficiency of the catalyst (CuO_x_/SiAl) was related to the presence of acidic sites on the support that are responsible for the competitive exchange of the Cu precursor via a chemisorption hydrolysis process. This exchange leads to the formation of isolated Cu(I) sites that were difficult to reduce. The selected best nanocatalyst was recycled two times, however the detection of metal leached from the surface suggested homogeneous copper species had a role in making this copper-oxide based catalyst a reservoir of active catalysts. 

**Scheme 3:**
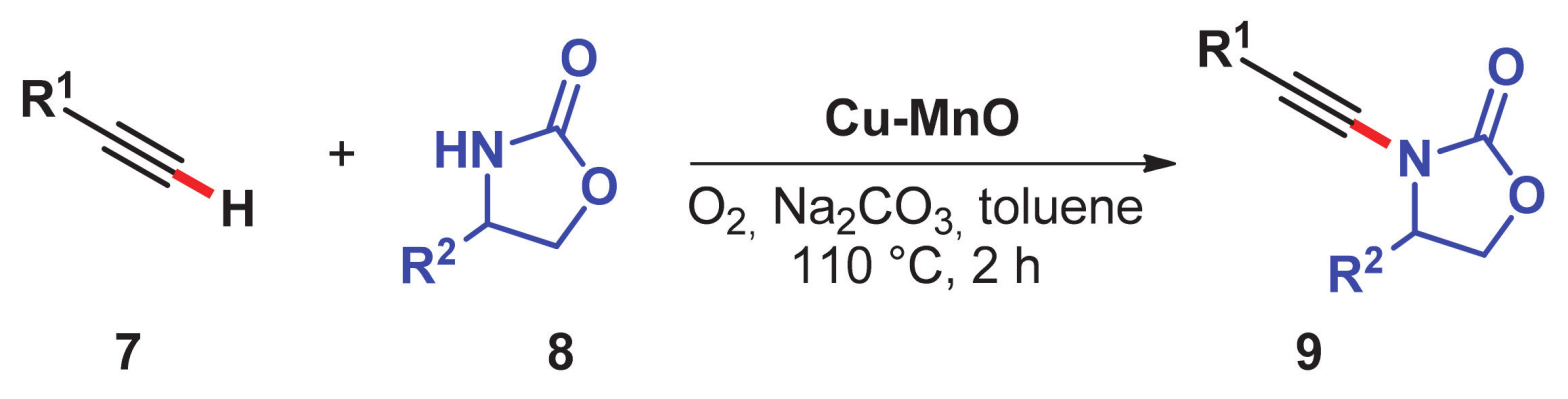
Oxidative alkynylation of **8** catalyzed by Cu-MnO.

In 2019, Gosh and coworkers reported an atom-economical approach to the synthesis of ynamide **9** via the oxidative alkynylation of amide using molecular oxygen as the sole oxidant [63]. The homogeneously dispersed Cu(II)/Cu(0) nanoparticles stabilized on spherical γ-MnO supports were efficient in the alkynylation of different oxazolidin-2-one derivatives **8** as well as in the reactions of *N*-methyl-*p*-toluenesulfonamide with aromatic and aliphatic alkynes **7** (Scheme 3). The authors found to be crucial the employment of Na_2_CO_3_ as a base and the presence of oxygen to obtain high yields in a short reaction time (2 h). The optimized conditions of the gram-scale reaction of phenylacetylene and oxazolidinone led to a 92% yield. In addition, the authors performed several kinetic experiments to give some insight about the mechanism of the reaction. By conducting the reaction in the presence of radical scavenger like BHT (butylated hydroxyl toluene) and TEMPO, yields comparable with those of the optimized reaction were obtained, suggesting that a radical pathway does not take place. The heterogeneous nature of the reaction was confirmed by a hot filtration test and the catalyst stability was confirmed by a low metal leaching (2 ppm), which allowed for the efficient recovery and reuse the Cu-MnO heterogeneous system for five consecutive reactions, maintaining similar productivity.

**Scheme 4:**
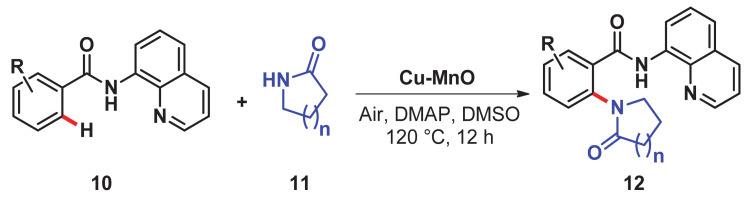
C–H amidation of **10** catalyzed by Cu-MnO.

Recently, the same group proposed a different protocol for the C–H amidation of 8-aminoquinoline benzamides **10** with different cyclic amides **11** using their Cu-MnO heterogeneous catalyst and air as oxidant (Scheme 4) [64]. The authors hypothesized that their MnO support may help the electron transfer pathway in the reductive elimination by working as an electron-transfer mediator (ETM). Indeed, the use of manganese oxide can easily reduce the oxidation gap potential between the reduced catalyst and molecular oxygen, allowing the use of oxygen or air as benign final oxidants [65,66,67,68]. The authors also found the use of DMSO as reaction medium and the use of DMAP as base and air as oxidants to be crucial for the entire efficiency of the process. The optimized protocol can be further scaled up to gram scale with comparable yield. Only a slight decrease in catalytic efficiency was observed during recovery and reuse, and the authors mainly attributed this to the sintering of the impregnated copper. Indeed, the authors excluded the possibility of reduced activity related to Cu leaching by measuring the metal content in solution (<1 ppm). In addition, the heterogeneity of the process was confirmed by a hot filtration test. Additional experiments to better understand the reaction mechanism were also performed. The results obtained by carrying out the reaction in presence of BHT and TEMPO as radical scavengers avoided the presence of a radical pathway. The k_H_/k_D_ ratio of 1.04 suggests that C–H bond activation was not the rate-determining step. Moreover, after performing the reaction with different designed substrates, the authors concluded that the presence of the *N,N*-bidentate ligand was crucial, suggesting that the reaction proceed through dehydrogenative amidation via C–H activation. The *ortho*-selectivity of C–H amidation is related to the presence of 8-amino quinoline as directing group.

## 3. C–H Amination

**Scheme 5:**
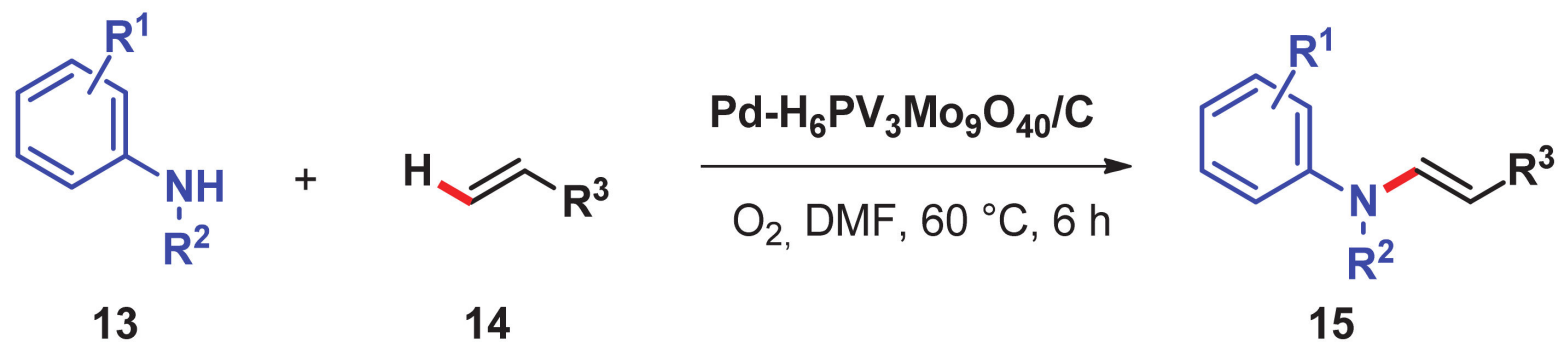
Oxidative C–H amination of acrylates **14** catalyzed by Pd-POM/C.

Among the C–H amination processes, Ying and coworkers developed in 2011 a Pd(II)-based nanocatalyst for the oxidative amination of acrylates **14** (Scheme 5) [69]. The catalyst is a Pd-polyoxometalate (POM) supported on a carbon named Pd-H_6_PV_3_Mo_9_O_40_/C, which showed superior catalytic activity in comparison to Pd-POM/C and also Pd/C. The authors found DMF to be the best medium, and that the presence of O_2_ was crucial for the oxidative process between diphenylamine and butyl acrylate. The nanocatalyst was recovered and reused for three consecutive runs with the addition of fresh H_6_PV_3_Mo_9_O_40_ after each cycle. Indeed, the authors measured 70% of POM lost after the recovery of the catalyst even if no change in the morphology of the Pd NPs was detected from TEM analysis. XPS spectra confirmed the presence of Pd(II) on the catalyst although after hot filtration test an increase in conversion was detected (from 30% to 45%) suggesting the presence of leached Pd, which acted as catalyst.

**Scheme 6:**
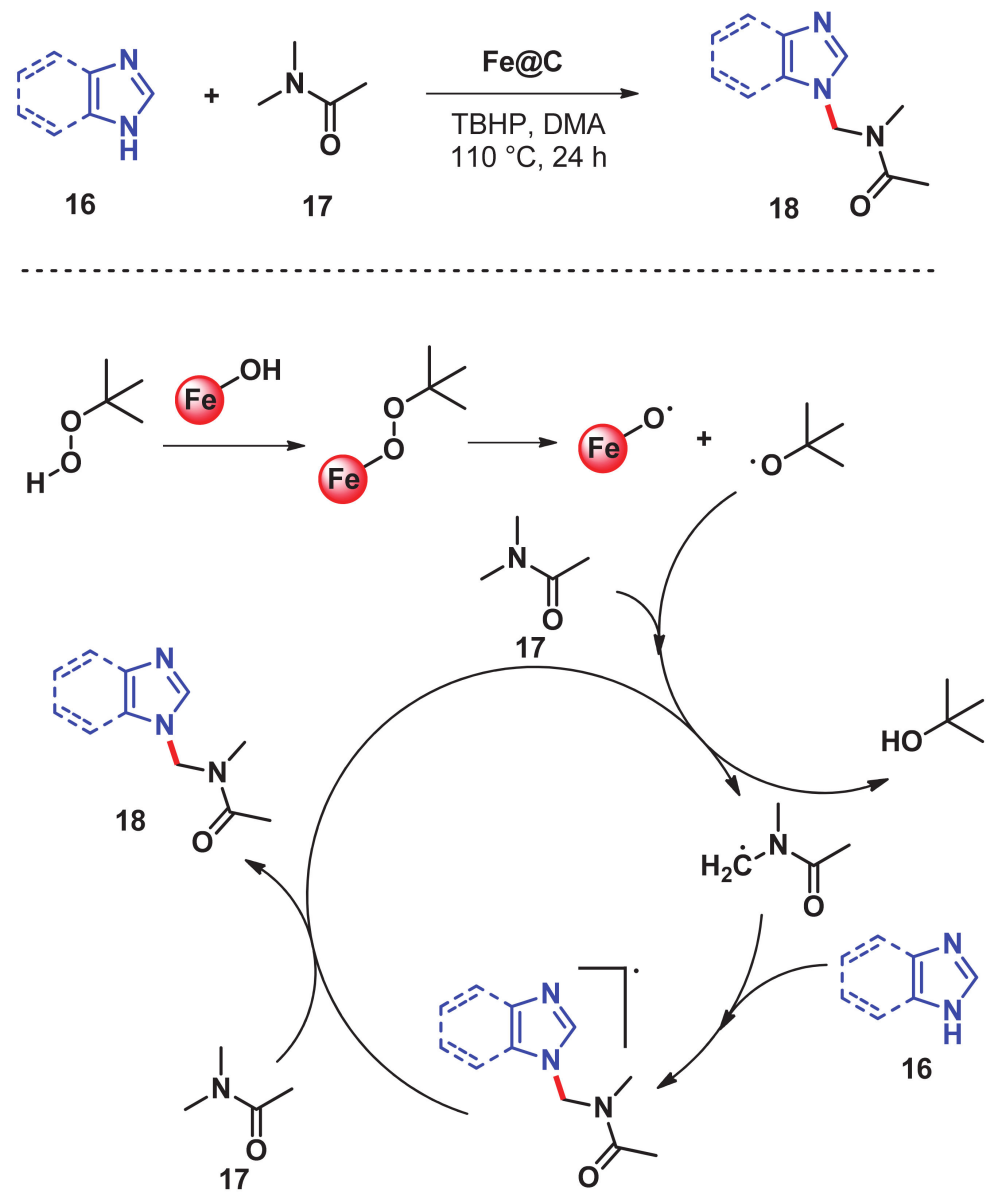
Oxidative coupling of **16** catalyzed by Fe@C.

In 2017 Gracia, Dhakshinamoorthy and coworkers reported the direct oxidative coupling of aromatic N–H compounds with different amides (Scheme 6) [70]. The authors investigated the influence of different ratios of Fe and Co nanoparticles embedded in a turbostratic graphitic carbon matrix and found a linear dependence of the enhanced catalytic efficiency with the decrease of cobalt percentage. With the selected Fe@C catalyst, the authors found as optimal reaction conditions the use of TBHP in the presence of an excess of dimethylacetamide (DMA) that acts both as compound for amination and as a reaction medium at 110 °C. After filtering the heterogeneous catalyst, less than 1% of the initial amount of Fe was detected in solution. To give more insight about the mechanism, experiments with homogeneous Fe species in different oxidation states were performed. In these conditions, product **18** was detected in <5%, confirming that the reaction did not proceed with the metal released in solution. However, after filtering the catalyst, the reaction did not completely stop. The reaction was then conducted in presence of TEMPO, leading only to the DMA–TEMPO adduct. By reacting this adduct without the presence of a Fe NP catalyst, a C–N coupling product was observed, suggesting that the role of Fe catalyst was as an initiator of TBHP decomposition. The catalyst was efficiently recovered and reused for five consecutive runs without changes in the reaction rate during the recycle. Indeed, the TEM images of the recovered catalyst are comparable with the images obtained with fresh Fe@C material. Both the Fe NP dimensions and C matrix morphology remained unaltered after the reaction.

Different C–H amination processes have been developed with the use of copper based nanocatalysts, employing as nitrogen sources primary [71,72] or secondary [64,72,73,74] amines, as well as ammonia [75].

**Scheme 7:**
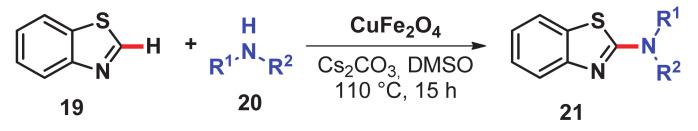
C–H amination of **19** catalyzed by CuFe_2_O_4_.

In 2014 Nageswar and coworkers reported the synthesis of 2-substituted benzothiazoles **21** through direct C–H amination catalyzed by magnetic copper ferrite nanoparticles (Scheme 7) [73]. The air-stable CuFe_2_O_4_ nanocatalyst can be easily separated from the reaction mixture thanks to its magnetic properties. This feature allowed the authors to efficiently recover and reuse the catalytic system for four consecutive runs without any loss in conversion. Furthermore, XRD, TEM, and SEM analyses confirmed the stability of the copper ferrite nanocatalyst. However, the authors did not envisage any mechanistic proposal regarding the direct C–H amination.

**Scheme 8:**
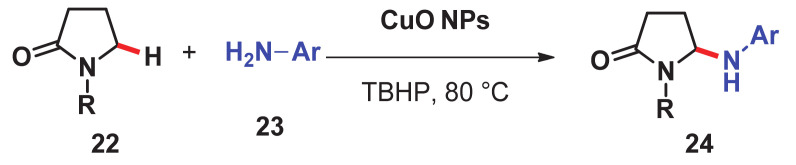
C–H amination of **22** catalyzed by CuO NPs.

During the same year, Kantam and coworkers proposed the synthesis of *N*-aryl-γ-amino-γ-lactams **24** from 2-pyrrolidinone **22** and aromatic or heteroaromatic amines **8**, catalyzed by CuO NPs (Scheme 8) [71]. The authors tested different copper-based catalysts, both homogenous and heterogeneous, and found CuO NPs to be the best catalytic system in the presence of TBHP as an oxidant. Due to the possibility of generating radicals using CuO NPs in combination with TBHP, the authors carried out the reaction in the presence of TEMPO and no conversion was detected under these conditions. This result led the authors to propose a single electron transfer mechanism (SET) to afford iminium intermediates after the generation of a radical by *tert*-butoxyl radicalic abstraction of the γ-proton of **22**. The catalyst was reused without loss in efficiency. XRD analyses revealed that no change in morphology occurred after the use of the CuO NPs.

**Scheme 9:**
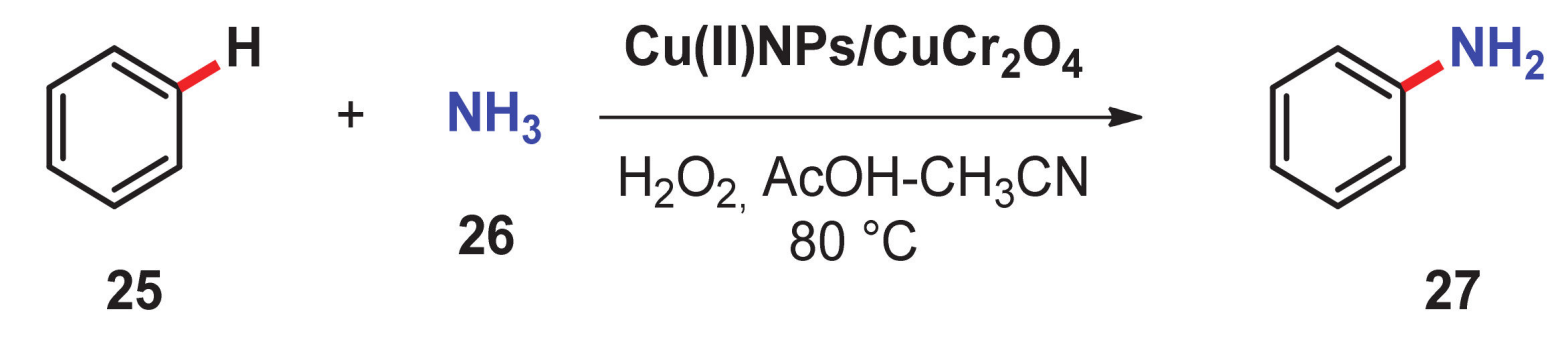
Oxidative C–H amination of **25** catalyzed by Cu(II)-CuCr_2_O_4_.

A different protocol for the oxidative amination of benzene **25** to aniline **27** promoted by a Cu(II)-based nanocatalyst in the presence of H_2_O_2_ was developed by Bal and coworkers (Scheme 9) [75]. The authors used a bimetallic copper-chromium oxide as a support for Cu(II) NPs (Cu(II)-CuCr_2_O_4_) and tested different reaction conditions to obtain the best selectivity in **27** with the lowest amount of phenol formed as side-product. The catalytic activity remainedc unchanged for up to five consecutive runs and no metal leaching was detected in solution, suggesting the heterogeneous nature of the catalyst. In addition, the change in conversion during the experiment in the presence of TEMPO as a radical scavenger led the authors to propose a reaction mechanism which involves the formation of NH_2_OH and a subsequent reduction to protonated amino radicals. Different catalytic tests were conducted by varying the ratio between NH_3_ **26** and H_2_O_2_, proving that the reaction path starts with the C–H bond activation of benzene in presence of H_2_O_2_.

**Scheme 10:**
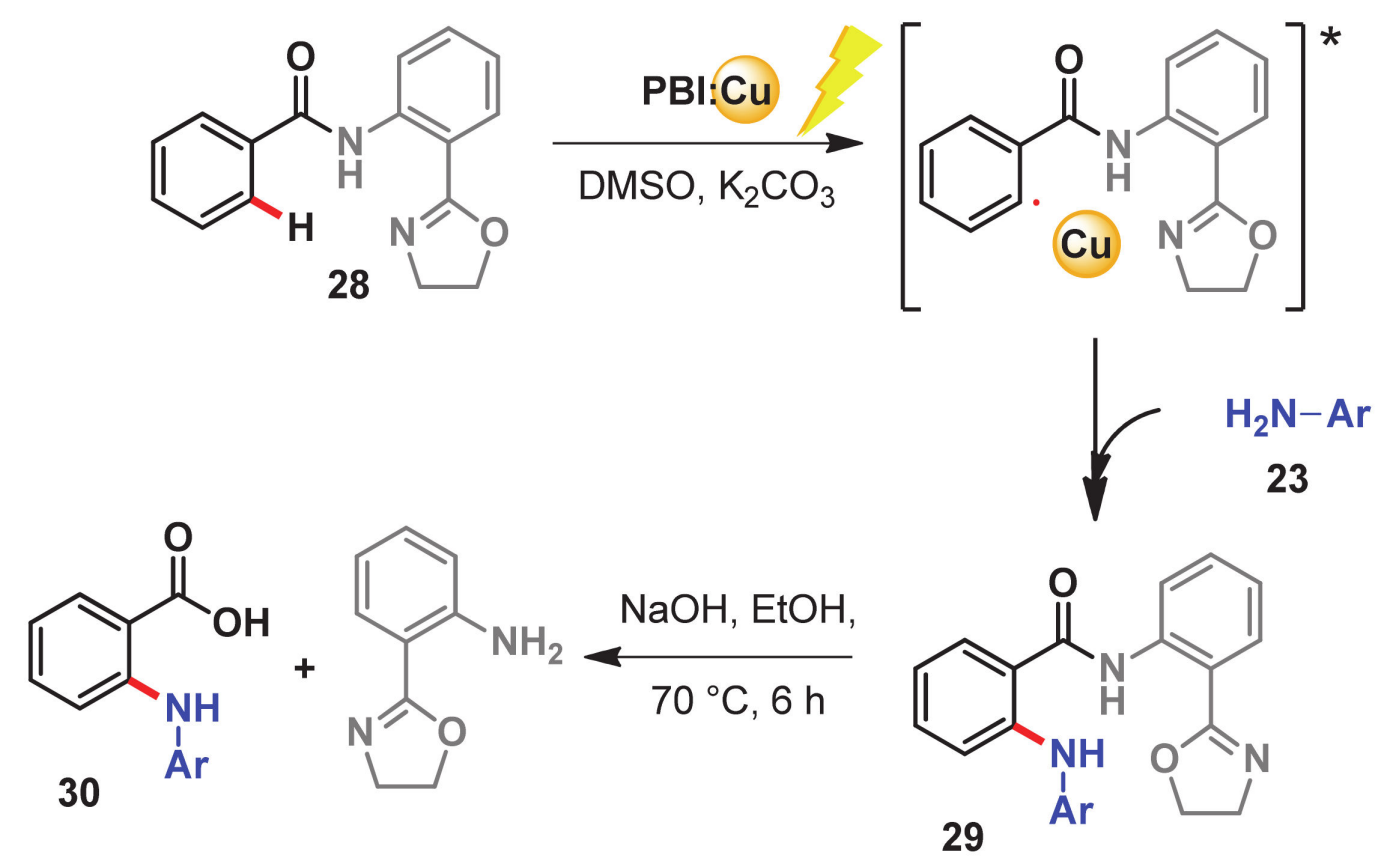
C–H amination of **28** catalyzed by PBI:Cu NPs and subsequent directing group cleavage.

Bhalla and coworkers proposed a supramolecular ensemble of a perylene bisimide (PBI) derivative and Cu NPs, generated in situ as a photocatalyst for direct *ortho* C(sp^2^)–H amination of benzamide derivative **28** [72]. The authors optimized the reaction conditions by employing *p*-nitro aniline and found that DMSO as medium and the presence of air gave the best conditions for the reaction (Scheme 10). The *ortho* C–H activation could be promoted by a single-electron transfer mechanism (SET) assisted by the electrons on the copper nanoparticle surfaces, which led to the free radical intermediates. A similar mechanism was proposed by the authors for the *ortho*-alkynylation of **28** catalyzed by the PBI:Cu NP photocatalyst [72]. The catalyst showed great performance with various anilines **23** as well as heterocyclic amines, morpholine, pyrrolidine, and piperidine. The PBI:Cu NPs were recycled for up to three consecutive runs. Moreover, in a second step the authors performed directing group cleavage, obtaining the corresponding carboxylic acid **30** in high yield.

**Scheme 11:**
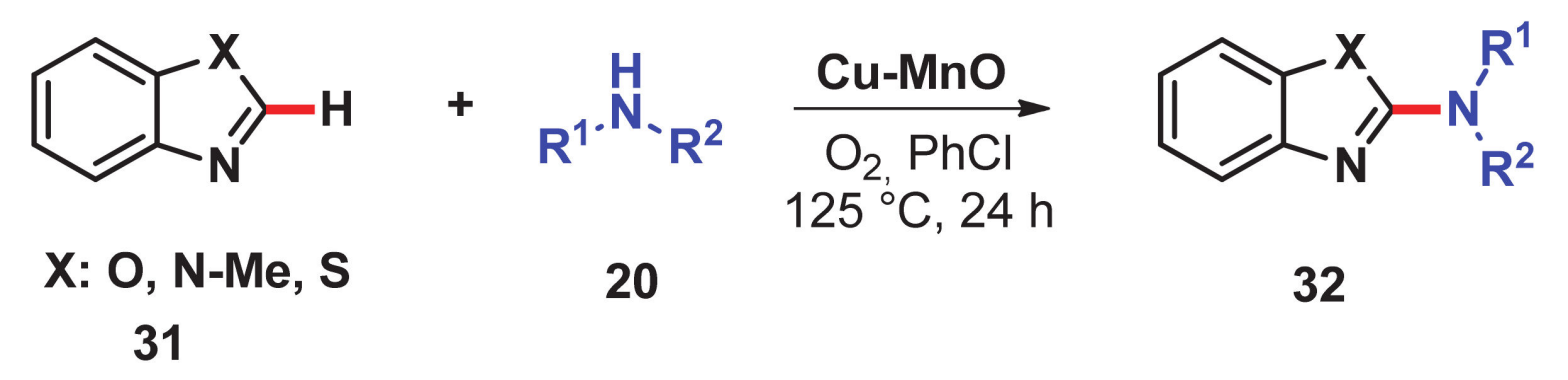
C–H amination of **31** catalyzed by Cu supported on lotus shaped MnO.

In 2017 Gosh, Panda, and coworkers reported the oxidative C–H amination of different azoles **31** catalyzed by copper and supported on a lotus-shaped manganese oxide heterogeneous catalyst (Scheme 11) [74]. The designed catalyst was able to overcome the limitations of their previously reported γ-MnO_2_ catalytic system that showed good performance only with benzoxazole derivates [76]. The authors prepared and tested several catalytic systems by varying the loading of Cu from 2.5 to 10 wt% in order to enhance the catalytic efficiency by the incorporation of copper into manganese oxide [74]. Only in the 10 wt% was Cu-MnO observed, with peaks associated to both metallic Cu and CuO in the XRD analysis, while no CuO was detected from 2.5 to 5 wt% of Cu-MnO catalysts. The optimal results in the oxidative amination of benzothiazole with morpholine were recorded using 5 wt% Cu-MnO in the presence of oxygen as an oxidant. The catalyst was also efficient in the C–H amination of *N*-methyl benzimidazoles and superior in comparison with γ-MnO_2_ when benzoxazoles were used as substrates. Although the copper in solution after the reaction was lower than 1 ppm, a decrease in catalytic efficiency was detected together with a decrease of surface area during the recovery and reuse for five consecutive runs. The authors ascribed this loss in efficiency to the sintering of Cu NPs.

**Scheme 12:**
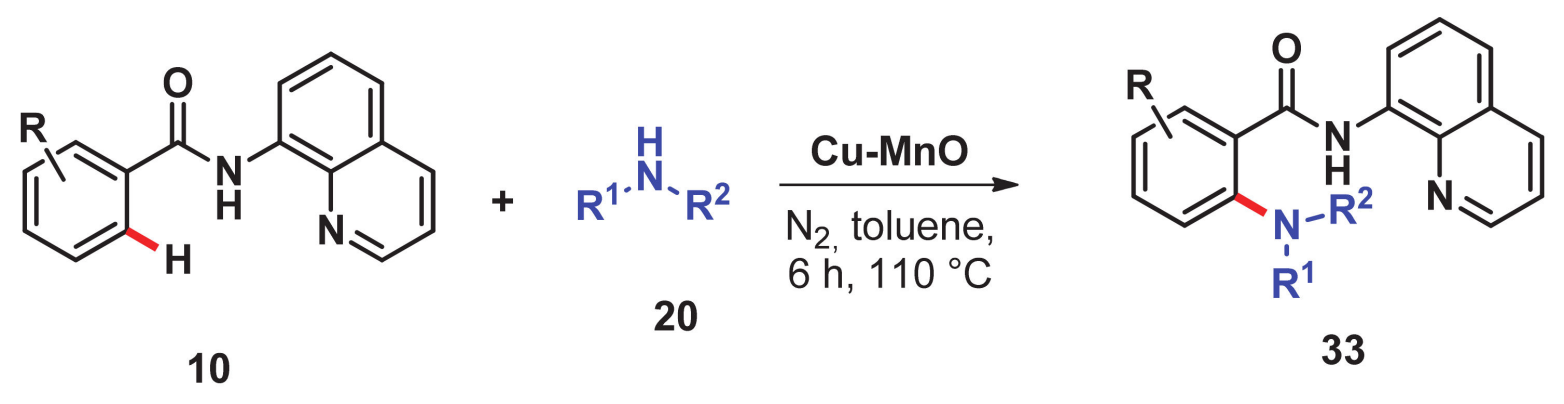
C–H amination of **10** catalyzed by Cu-MnO.

Very recently, the same group employed copper-supported on spherical MnO in the C–H amination of 8-aminoquinoline benzamides **10** (Scheme 12) [64]. In comparison with the results previously described for the amidation reaction, the direct amination proceeds under milder reaction conditions. After 6 h at 100 °C, different products were obtained in excellent yields without the addition of any additives. Only the primary amine and indole were not compatible with the optimized reaction conditions. However, when the reaction was scaled up to gram scale, a comparable yield was recorded in the C–H amination of **10** with 4-methylpiperidine. For the amidation, the Cu-MnO was efficiently recycled for five consecutive runs with negligible loss in efficiency associated with the sintering of the impregnated copper.

**Scheme 13:**
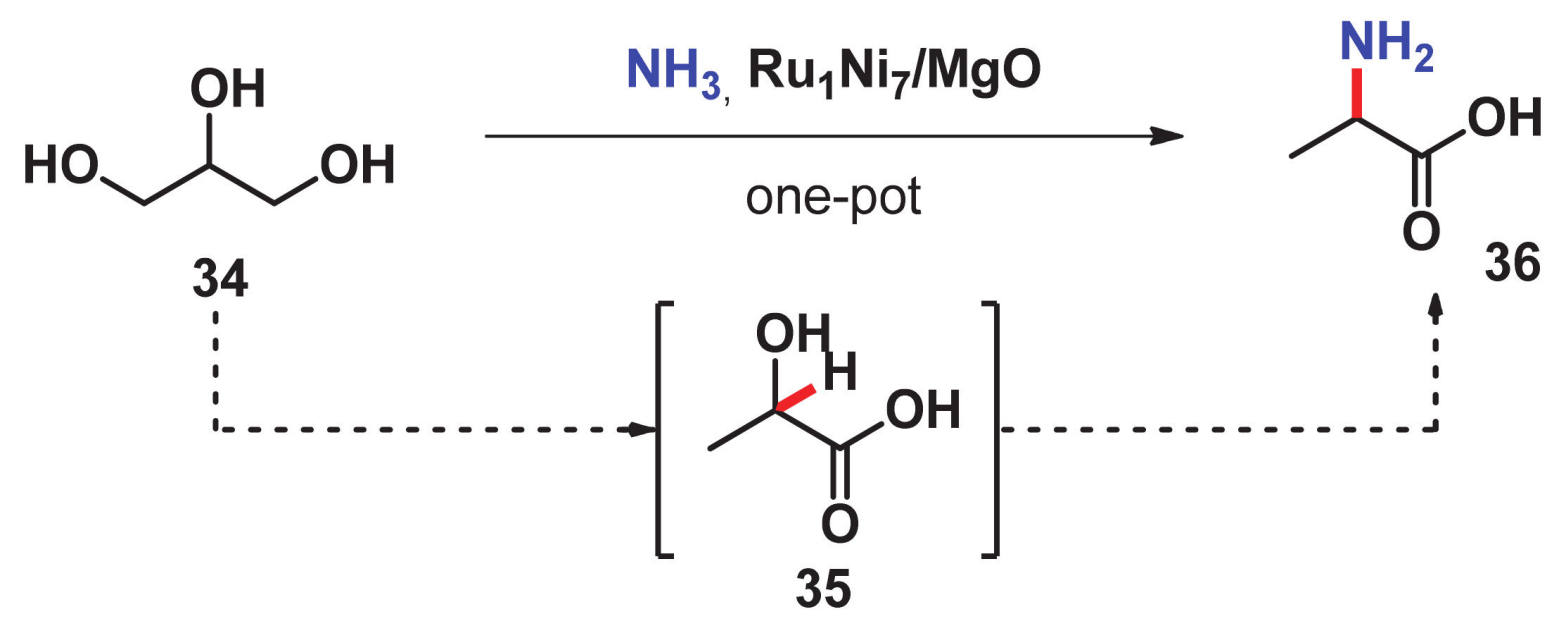
Alanine production via C–H amination from glycerol waste.

Yan and coworkers developed a bimetallic RuNi nanocatalyst supported on MgO for the one-pot conversion of glycerol **34** to alanine **36** through the amination of the lactic acid intermediate **35** via C–H activation (Scheme 13) [77]. The authors prepared and tested different bimetallic Ru-based nanocatalysts by changing the metal, the ratio between two metals, the support, and the temperature of calcination. An increase in catalytic activity was observed for RuNi NPs in the molar ratio 1:7, with a range of 500–600 °C as the ideal temperature for calcination using MgO as a support. The authors attributed the effect of the support to its chemical stability under the basic reaction conditions, while the temperature of calcination influences the nature of the Ru species as well as the presence of Ni. Indeed Ru_1_Ni_7_/MgO presents much more similarity with metallic Ru than does Ru/MgO, as deduced from XPS analysis. After kinetic studies, the author suggested that the amination of **35** to **36** via C–H activation was the rate determining step. In order to explain the role of Ni in the catalytic cycle, the authors conducted DFT calculations that suggested that Ni can enhance the catalytic activity of Ru in the conversion of lactic acid to alanine by binding the oxygen alkoxide atoms. The catalyst was tested on 56% purity crude **34** and was recovered and reused three times. During the third run, some decrease in activity was observed, and the authors suggested that this could be related to some increase of NP size after recovery. The oxidation state of the two metals remained unchanged.

## 4. C–N Bond Formation in the Synthesis of Heterocycles

**Scheme 14:**
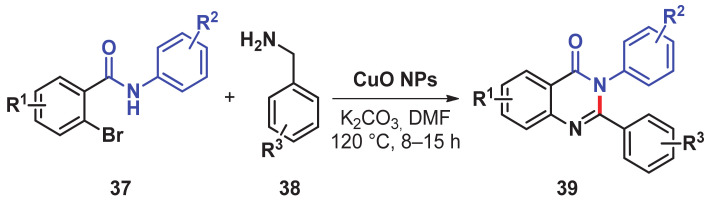
Oxidative C–H amidation for the synthesis of 2,3-disubstituted quinazolinones **39** catalyzed by CuO NPs.

Particularly intriguing is the preparation of widely useful heterocycles exploiting direct C–N bond formation. An example is given by the intramolecular oxidative C–H amidation process proposed by Patel and coworkers for the synthesis of 2,3-disubstituted quinazolinones **39** (Scheme 14) [78]. Using CuO nanoparticles, several quinazolinones were obtained from different *orto*-halobenzamides **37** and benzylamines **38** in DMF under air via an Ullman coupling followed by an oxidative intramolecular C–H amidation. To further confirm the proposed mechanism, the authors performed the reaction under inert atmosphere, obtaining only Ullman’s product. The latter was placed under optimized reaction conditions, in the presence of air, affording the desired product and confirming the presence of this intermediate during the reaction. After the recovery and reuse of the CuO nanocatalyst, a slight decrease in catalytic efficiency was observed due to a change in the morphology of the CuO material after the recovery. Indeed, TEM images of the used catalyst showed the presence of particle aggregates formed during the reaction. 

The majority of processes involved in the direct synthesis of heterocycles are generally catalyzed by Pd-based nanocatalysts.

**Scheme 15:**
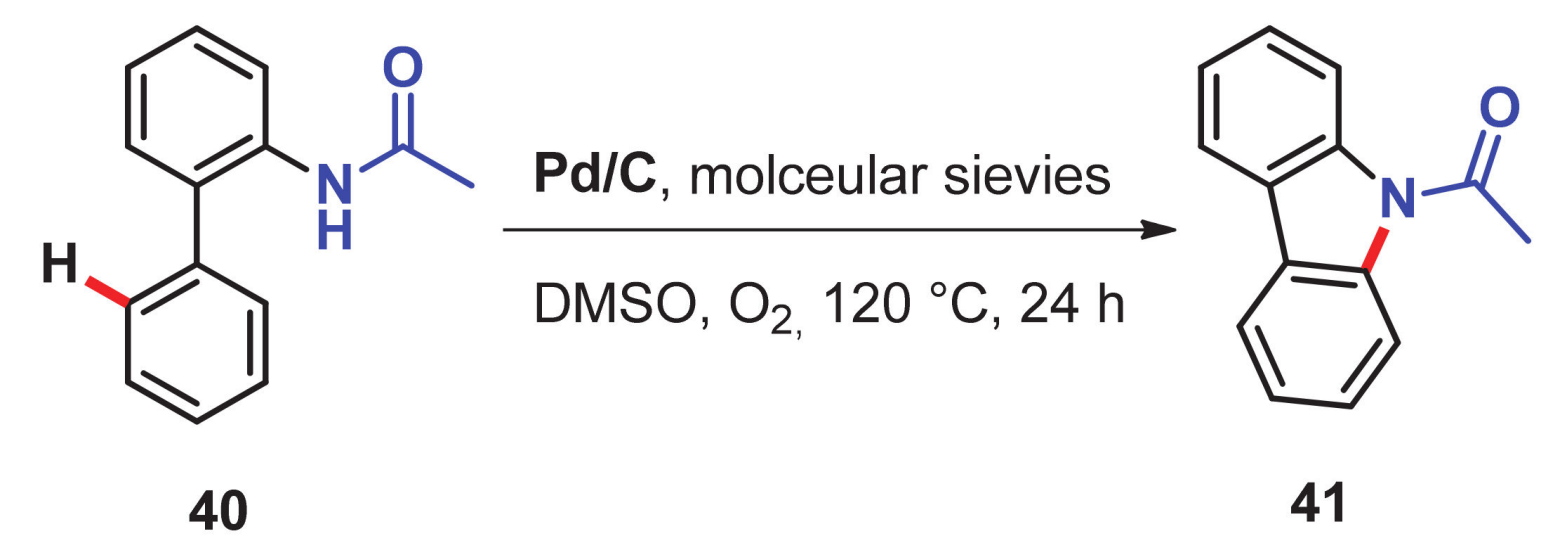
Intramolecular C–N bond formation for the synthesis of carbazoles catalyzed by Pd/C.

In 2014 Ying and coworkers developed eight different palladium NP-based catalysts for the synthesis of carbazoles **41** via intramolecular C–H amination (Scheme 15) [79]. The authors investigated the role of the support in Pd stabilization, highlighting the dependence of catalyst reactivity on the interactions between Pd and the support. After testing Ag-Pd/C, Ag@Pd/C, Ag_2_S@Pd/C, Pd/C, Pd/CeO_2_, Pd/TiO_2_, Pd/Al_2_O_3_ and Pd/SiO_2_ in DMSO as a reaction medium, the highest catalytic activity was that of Pd/C. This material was then selected for further optimization. The authors found the use of 4 Å molecular sieves crucial to remove the only byproduct, water, formed during the reaction using O_2_ as oxidant. Despite the good tolerability of the Pd/C catalyst for different substrates, during the recycle experiments the conversion dropped by 12% and 50%, respectively for the two subsequent recycles. The drop in catalytic efficiency was explained by TEM analyses that revealed an increase of the particle size distribution from 2–5 nm to 20–30 nm. However, the Pd 3d peaks from XPS analyses before and after the C–H amination process showed no significant modification of the surfaces. By performing a series of hot filtration experiments, with and without oxygen, the authors proposed a homogeneous nature of the catalysis driven by both O_2_ and DMSO. Indeed, after the filtration of solid catalyst the reaction proceed only in presence of oxygen. The role of oxygen reveals to be crucial for the solubilization of Pd(II) active species, probably as Pd-DMSO complexes. The same behavior was also revealed when the CeO_2_, TiO_2_, and Al_2_O_3_ Pd-supported nanomaterials were employed. The Pd/C, Pd/CeO_2_, and Ag_2_S@Pd/C nanocatalysts presented a higher amount of Pd(II) species than the other materials. Among these, the inactive Ag_2_S@Pd/C had its Pd(II)-associated peaks shifted to lower values. The authors suggested that the different interactions between Pd(II) and the supports influenced catalytic efficiency, as it is related to the the dissolution of palladium in DMSO.

**Scheme 16:**
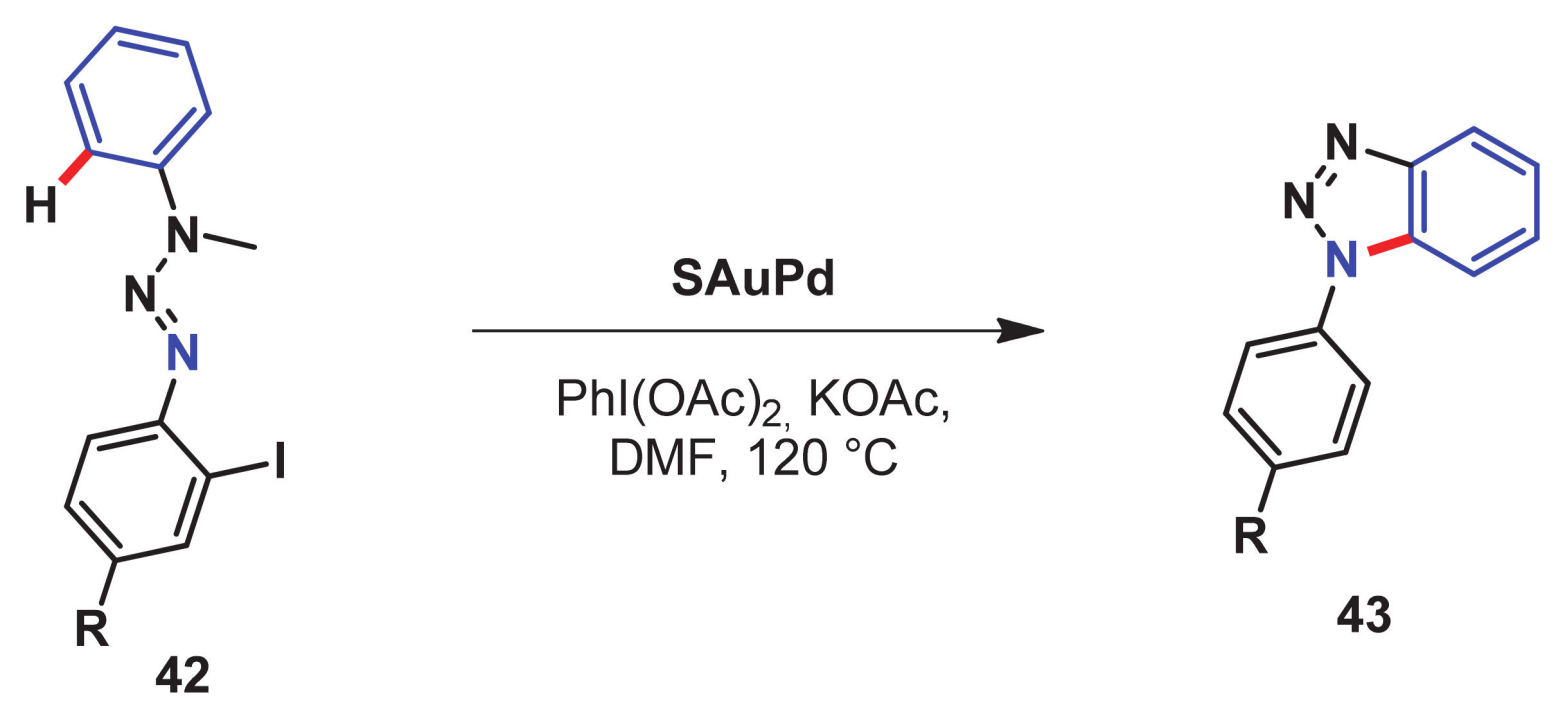
Synthesis of **43** via sequence of 1,7-palladium migration, cyclization, and dealkylation catalyzed by SAuPd.

During the same year, Arisawa and coworkers reported the synthesis of *N*-aryl-benzotriazoles **43** via a sequence of 1,7-palladium migration, cyclization, and dealkylation catalyzed by sulfur-modified gold-supported Pd NPs (SAuPd) (Scheme 16) [80]. The authors focused initially on the choice of the most adequate oxidant to promote the formation of Pd(II) species and found that PhI(OAc)_2_ combined with KOAc as a base and DMF as the medium to produce optimal reaction conditions. The protocol is applicable to different substrates; however, recycling or further tests to determine the mechanism and the nature of the catalysis were reported when compound **43** was tested as inhibitory of indoleamine 2,3-dioxygenase in vitro.

**Scheme 17:**
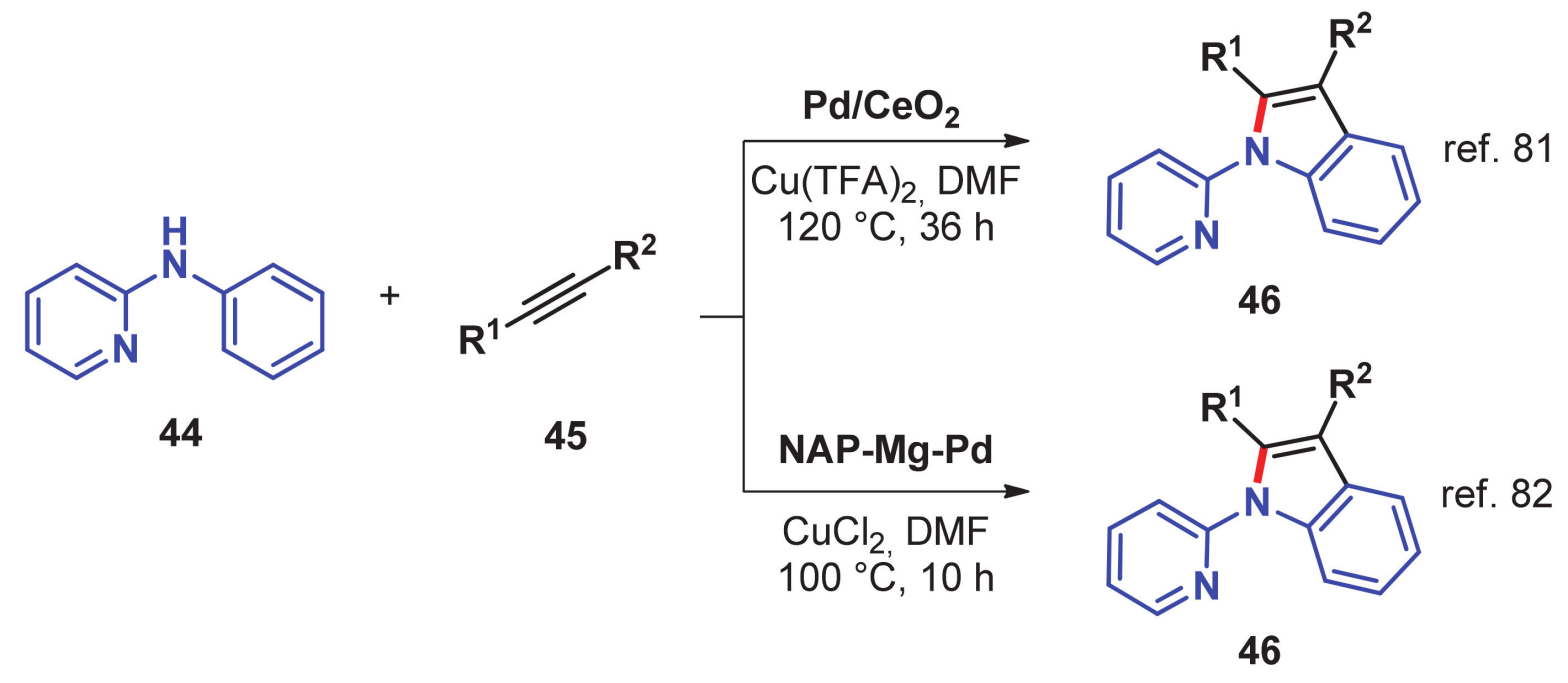
Synthesis of **46** catalyzed by NAP-Mg-Pd.

In 2014 Wu and coworkers developed an efficient protocol for the synthesis of the *N*-pyridyl indole **46** by coupling *N*-phenyl-2-aminopyridine **44** with disubstituted alkynes **45** (Scheme 17) [81]. An exhaustive screening of the catalyst was performed, varying both the metal and the support. Among the metals tested (Pd, Ru, and Pt), palladium gave the best results, while replacing the CeO_2_ support with TiO_2_ or Al_2_O_3_ gave poor results. With the selected Pd/CeO_2_ nanocatalyst, further mechanistic experiments were conducted, suggesting the heterogeneous nature of the catalysis. Indeed, both in the Hg-poisoning test and in the reaction conducted with homogeneous Pd(OAc)_2_ only poor conversion into product **46** was detected in the reaction mixture. However, during the recycling experiment, an important drop in conversion was observed in the second run. The competitive kinetic isotope experiment revealed that the C–H activation was not the rate-determining step.

One year later, Kantam and coworkers developed an efficient Pd-based heterogeneous catalyst for the same process (Scheme 17) [82]. The authors tested different heterogeneous Pd(0) NP-based catalysts and found the role of the nanocrystalline magnesium oxide support beneficial to the reaction using CuCl_2_ as oxidant. The developed catalyst (NAP-Mg-Pd(0)) was efficiently recovered and reused for four consecutive runs without loss in efficiency or Pd leaching in solution. Moreover, the comparison of TEM images between the fresh and spent catalysts showed no change in morphology after four cycles. However, further experiments about the reaction mechanism were not reported by the authors.

**Scheme 18:**
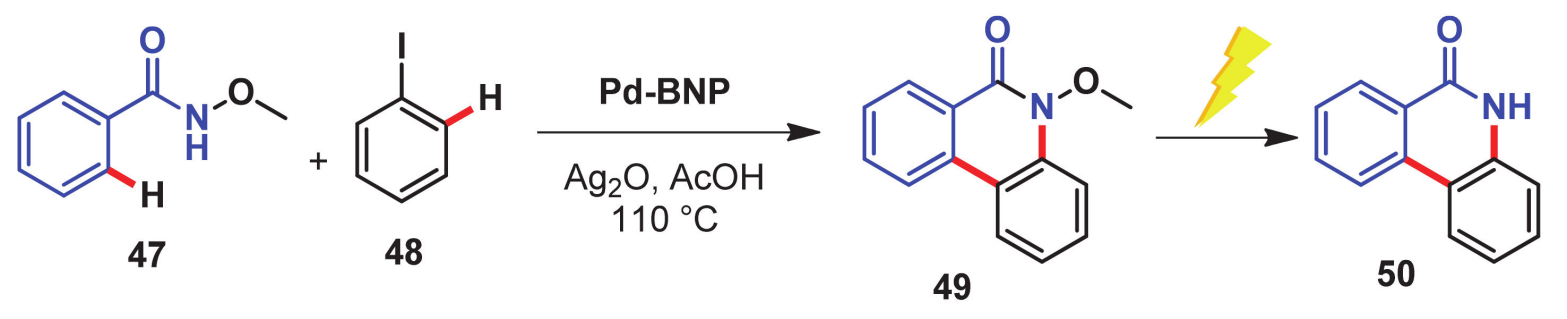
Synthesis of phenanthridinones catalyzed by Pd-BNP.

Recently, Sekar and coworkers developed the synthesis of phenanthridinones **50** via C–H activation of *N*-methoxy benzamides **47** and aryl iodides **48** (Scheme 18) [83]. The authors tested several Pd NPs using different stabilizers and commercially available Pd/C and Pd NP. The best reactivity was observed using binaphthyl for Pd NP stabilization (Pd-BNP) in combination with Ag_2_O as the oxidant and acetic acid as a solvent. In order to further investigate the mechanism, the authors performed different control experiments. In conclusion, they suggested that the mechanism starts with a C–H bond activation and selective C–C bond formation while the C–N bond formation occurs only at the end. In addition, the presence of the *N*-methoxy group was essential for the completion of the process, and specifically for the final C–N bond formation. Eventually, the methoxy group can be easily removed (45 min reaction time) in the presence of visible light. UVvis spectra demonstrated that no change in the morphology of the Pd NP occurred after the reaction. Moreover, hot centrifugation and Hg poisoning tests showed that no metal leaching was detected in solution. 

**Scheme 19:**
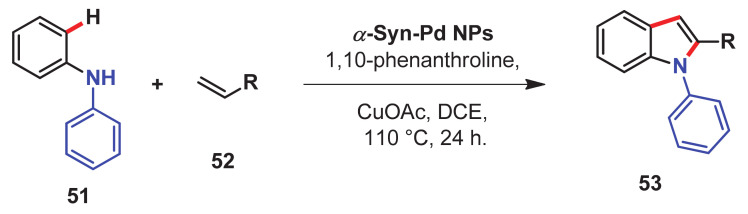
Synthesis of **53** catalyzed by *α-*Syn-Pd NPs.

A Pd NP catalyst was also developed by Maji, Maiti and coworkers and employed in the synthesis of 2-substituted *N*-aryl indoles **53** (Scheme 19) [84]. The catalyst consists of Pd NPs stabilized on α-Synuclein (α-Syn) fibrils showing good performance in the synthesis of **53** via triple C–H activation between the *N*-phenyl anilines **51** and the terminal alkenes **52**. The synergy between thes amyloid fibrils and Pd NPs was crucial, as proven by control experiments conducted in the absence of, or varying the support or the structure of, the fibril. Under the optimized conditions, the catalyst could be recovered and reused for three consecutive runs with no change in the morphology of the Pd NPs, while at the same time a slight modification in the amyloid fibril structure was observed. 

## 5. Conclusions and Future Outlooks

The direct construction of novel C–N bond is of great interest in different areas leading to an atom-economical strategy to access high-value products. The precious metals, generally used as homogenous catalytic systems, could possibly be replaced with more convenient heterogeneous systems that allow for an easy separation of the catalyst from the reaction mixture, the reuse of the catalytic system and a minimization of product metal contamination.

In this contribution, we focused on the employment of metal nanoparticles as heterogeneous catalytic systems. Metal choice and the selection of the most appropriate support are generally the pivotal parameters to define efficient nanocatalytic systems, and this is also true in the formation of C–N bonds via C–H activation. Indeed, catalyst design at nanometric size enhances the catalytic performance of otherwise less-reactive non-noble hearth-abundant metals. The preparation and use of MNPs can be also seen as a sustainable approach for the use of low-abundant noble metals in direct C–N bond formation and for eventual catalyst recovery.

The literature concerning C–H amidation reactions is dominated by protocols employing 3d-metal NP (Co and Cu) or their oxides (CuO), and the same goes for C–H amination (Fe, Cu and CuO). Rare are the examples of Pd- or Ru-based catalysts. In the exploitation of C–N bond formation for the synthesis of heterocycles (last section), the majority of the contributions report the use of Pd NP catalysts.

When using a solid catalyst, the definition of the actual active mechanism may be non-trivial. When possible in the discussed examples, the experiments performed to determinate the rate-determining step and the role of MNPs in the catalytic cycle were discussed.

In the realm of C–N bond formation via C–H functionalization catalyzed by MNPs, there are few examples reporting the use of photocatalysis to improve catalyst performance. To the best of our knowledge, the combination of nanocatalysis with other enabling technologies has not been reported. While MNP-catalyzed direct C–N bond formation can benefit from the use of flow chemistry, microwave or ultrasound activations can also help to scale-up the procedure. The engineering of nanocatalysts and their synergy with novel methodologies are expected to improve this process from a sustainability point of view.
